# EHMTI-0087. Use of oxicodone/naloxone extended release for menstrual migraine

**DOI:** 10.1186/1129-2377-15-S1-G27

**Published:** 2014-09-18

**Authors:** C Mostardini, G Pauletti

**Affiliations:** 1 Neurology Department, Giovan Battista Grassi Hospital, Roma, Italy

## Introduction

Menstrual migraine is a big challenge for the migraine expert. Severity and duration of hormonal stimulation determines the gravity and duration of menstrual attack.

Various prevention therapy try to alleviate these attacks, usually long half-life triptans and-or anti-inflammatory drugs, combined with caffeine, magnesium, but however is very difficult to find the right drug in particular in women with uterine fibroids or endometriosis.

## Aims

To support this limited class of patients, we considered to use the Oxycodone/Naloxone Extended Release (O/N-ER) for its favourable mechanism of action on neuropathic pain, long half life and well documented gastrointestinal safety. Although the drug has an indication for chronic pain, many reports in literature support its use in acute pain.

## Methods

Recruitment is only for patients non-responders to classical preventive therapy. Will be collected informed consent, headache diary and a satisfaction questionnaire on therapy, those data will compared with the one recorded in the previous quarter, pharmacoeconomic analysis will provided.

Patients will use O/N-ER exclusively for menstrual attacks and for three consecutive periods, with the possibility of use a triptan at the start of attack followed by O/N-ER bid for 3-5 days.

## Results

Of the 8 patients that have completed the study (12 patients are ongoing in the trial), 70% of the patients showed a mean reduction in intensity of attacks from 8 to 4 on a NRS scale, grade of satisfaction was 8/10, without significant side effects. More data will come from the analysis of the entire group of the enrolled patients.

No conflict of interest.

